# Spatiotemporal Distribution and Clinical Characteristics of Zoonotic Tuberculosis, Spain, 2018–2022

**DOI:** 10.3201/eid3107.250031

**Published:** 2025-07

**Authors:** Álvaro Roy, Diana Gómez-Barroso, Elena Cruz-Ferro, Ana Fernández, Isabel Martínez-Pino, María del Henar Marcos, Isabel Ursúa-Díaz, Susana Miras, Nuria Echave, Evangelia Ouranou, Beatriz Romero, Laura Herrera-León, Zaida Herrador, Study Group on Zoonotic Tuberculosis

**Affiliations:** Instituto de Salud Carlos III, Madrid, Spain (Á. Roy, D. Gómez-Barroso, N. Echave, L. Herrera-León, Z. Herrador); CIBER in Epidemiology and Public Health, Madrid (D. Gómez-Barroso, I. Martínez-Pino, L. Herrera-León, Z. Herrador); Consejería de Sanidad Xunta de Galicia, Santiago de Compostela, Spain (E. Cruz-Ferro, I. Ursúa-Díaz, S. Miras); Consejería de Salud y Servicios Sanitarios Gobierno del Principado de Asturias, Oviedo, Spain (A. Fernández); Consejería de Sanidad Junta de Castilla y León, Valladolid, Spain (I. Martínez-Pino, M.H. Marcos); Aristotle University of Thessaloniki, Thessaloniki, Greece (E. Ounarou); VISAVET Health Surveillance Centre, Madrid (B. Romero); Complutense University of Madrid Faculty of Veterinary Medicine, Madrid (B. Romero)

**Keywords:** tuberculosis and other mycobacteria, Mycobacterium bovis, Mycobacterium tuberculosis, Mycobacterium caprae, zoonotic tuberculosis, One Health, zoonoses, bacteria, respiratory infections, Spain

## Abstract

Zoonotic tuberculosis (zTB) is a communicable disease that has major effects on both human and animal health. Spain reports the highest number of zTB cases in humans annually in the European Union. We describe the epidemiology of human cases of zTB caused by *Mycobacterium bovis* and *M. caprae* in Spain during 2018–2022. The incidence of *M. bovis* infection compared with *M. tuberculosis* infection was higher in patients who were native-born (adjusted odds ratio [aOR) 2.32, 95% CI 1.44–3.82), HIV-negative (aOR 3.39, 95% CI 1.24–14.0), or had extrapulmonary forms of TB (aOR 2.20, 95% CI 1.46–3.28). The spatial pattern differed by *M. tuberculosis* complex species; we identified 3 significant clusters of *M. bovis* and 1 of *M. caprae* in bovine TB–free regions. Our results show the importance of including animal and human data on circulating zoonotic pathogens under the One Health umbrella.

Tuberculosis (TB) in humans is caused by mycobacterial species of *Mycobacterium tuberculosis* complex (MTBC), mainly *M. tuberculosis*. Zoonotic TB (zTB) is a form of human TB caused by closely related species of mycobacteria, such as *M. bovis* and *M. caprae*, that are normally isolated from domestic or wild mammals, their natural hosts (*1*). *M. bovis* is the second most common cause of TB in humans and was estimated to be responsible for ≈1.4% of human TB cases worldwide (*2*). Cattle are the main reservoir of *M. bovis*, known as bovine TB, but *M. bovis* also causes TB in other animal species, including wildlife (*1*). *M. caprae* also has a notable importance in zTB incidence in Spain (*3*); it is the main causal agent of TB in goats and has also been reported in domestic and wild animals (*4*). It differs from *M. bovis* in that it is evolutionarily older and that most of the reported human cases are mainly concentrated in a few countries in central and southern Europe (*5*).

Transmission of the agents of zTB to humans is mainly indirect and usually occurs through consumption of contaminated milk and other dairy products that have not been subjected to sanitization processes. More rarely, it may result from consuming contaminated raw or undercooked meat. Cases of direct airborne transmission from animals or animal products to humans, as well as person-to-person transmission, have also been reported (*6*,*7*). Current molecular techniques suggest potential airborne transmission between animals and humans, until recently unclear and debatable (*8*); this possibility is particularly important in professions with a higher risk for exposure, such as farmers, veterinarians, hunters, or slaughterhouse workers (*9*).

The implications of zTB go beyond human health; it also causes losses to the livestock sector through reduced meat and milk production and slaughter of infected animals and movement restrictions, and losses to the state through the costs of eradication programs (*10*). Although infection in cattle herds appears to be under control in most high-income countries through bovine TB eradication programs, complete elimination of the disease is complicated by the limitations of diagnostic tests and the maintenance of infection in wild and domestic reservoirs (*11*).

Spain is the European Union (EU) country that reports the highest number of zTB cases annually in humans (0.11 confirmed cases/100,000 population in 2023) (*3*). It has a herd prevalence of tuberculosis in cattle of 1.5%, although this percentage varies widely by region (*12*). Animal TB in the Iberian Peninsula is mainly found in cattle and goats, but it also occurs in wildlife, so we should consider maintenance communities rather than specific hosts as reservoirs of infection (*13*). To estimate the real burden of disease in Spain and thus be able to design and evaluate specific and concerted actions, the zTB public health problem must be addressed with a One Health approach. In this article, we describe the reported cases of zTB in Spain during 2018–2022, making a clinical and epidemiologic comparison with TB cases caused by *M. tuberculosis* in the same study period.

## Methods

### Study Design, Population, and Data Sources

Our study used epidemiologic data from the National Network of Epidemiologic Surveillance (RENAVE), hosted by the National Center of Epidemiology. We conducted a retrospective descriptive study using surveillance data on human zTB and TB cases caused by *M. tuberculosis* reported to RENAVE during January 1, 2018–December 31, 2022. The regional surveillance systems of the autonomous communities (CCAA) report individual data on suspected (meeting the clinical criteria), probable (meeting both clinical criteria and laboratory criteria for a probable case), and confirmed (meeting both clinical and laboratory criteria for a confirmed case) cases of TB through the national reporting electronic platform. The notification is made following the protocol agreed by all the members of RENAVE, who are representatives of the CCAA, Carlos III Health Institute, and Ministry of Health. Case definitions in our protocol are based on the EU case definitions, as published in the Official Journal of the European Union (Commission Implementing Decision [EU] 2018/945) (*14*,*15*).

For our study, and to improve data quality, we contacted all the regions of Spain comprising 17 CCAA and 2 autonomous cities and requested they check the information provided on zTB cases from 2018–2022 at the beginning of the study. This process enabled us to recover 56 cases of zTB in RENAVE that did not have information about the causal agent. We included populations officially residing in Spain, obtained from the National Institute for Statistics, in the denominator (at national, provincial or municipal level depending on the analysis). We calculated incidence of zTB and TB cases per year and annual incidence rates per 100,000 population per year by age group and sex and by 1 million population per year by province. For the spatial cluster analysis, we calculated incidence at the municipal level.

### Data Analysis

After excluding imported cases, we conducted a retrospective descriptive analysis on the main sociodemographic and clinical characteristics: age, sex, country of birth, primary location of disease, HIV status, hospitalization, and treatment outcome. For the descriptive analysis of the qualitative variables, we calculated frequencies and percentages. We explored differences in the characteristics of zTB and TB (*M. tuberculosis*) patients with bivariate analysis; we used χ^2^ for multiple comparisons of qualitative variables and Mann-Whitney U tests with Bonferroni adjustment for quantitative variables. We estimated crude odds ratios (ORs) and adjusted ORs (aORs) and 95% CIs; we included those variables with p<0.1 in a logistic regression model. We used R version 4.4.0 (The R Project for Statistical Computing, https://www.r-project.org) for analyses. 

To assess temporal and geographic patterns, we calculated zTB and TB incidence per 100,000 population by year and per 1 million population by province using population denominators obtained from the National Statistics Institute. We analyzed temporal trends using linear and joinpoint regression analysis (Joinpoint version 4.9.1.0, https://surveillance.cancer.gov/joinpoint). We used purely spatial Poisson probability model of SaTScan software version 10.2.4 (https://www.satscan.org) to analyze geographic clusters of *M. bovis* and *M. caprae* incidence at the municipal level during 2018–2022. We restricted the spatial window to a maximum radius of 25 km, representing the mean distance between municipalities in Spain. We created maps using QGIS version 3.36.0 (QGIS, https://qgis.org) and ArcGIS version 10.8.1 (ESRI, https://www.esri.com).

### Ethics Considerations

We obtained data from the National Statistics Institute through open data access; thus, no ethical approval or informed consent was required to conduct data analysis, in accordance with the Spanish Human Research Act. All data were anonymized and deidentified. The research ethics and animal welfare committee at the Health Institute Carlos III approved the overall project (CEI PI 40_2023).

## Results

### Characteristics of Zoonotic TB Cases

A total of 18,852 autochthonous TB cases were reported to RENAVE during 2018–2022; of those, 6,098 (32.3%) cases had information on the species of MTBC. *M. tuberculosis* was identified in 5,849 TB cases, *M. bovis* in 218 cases, and *M. caprae* in 31 cases. The male-to-female ratio in *M. caprae* cases was 4.2, more than twice the ratio for *M. bovis* (1.9) and *M. tuberculosis* (1.8) cases.

The percentage of cases born in another country was 19.4% (6/31) for *M. caprae*, 24.8% (54/2,018) for *M. bovis*, and 36.5% (2,133/5,849) for *M. tuberculosis* (Table 1). The aOR of *M. bovis* infection among confirmed TB cases was more than twice as high for those born in Spain than for those born outside (aOR 2.32; 95% CI 1.44–3.82; Table 2).

**Table 1:** Characteristics of patients with confirmed *Mycobacterium caprae*, *M. bovis*, and *M. tuberculosis* infection in study of tuberculosis, Spain, 2018–2022*

Category	*M. caprae,* no. (%), n = 31	*M. bovis,* no. (%), n = 218	*M. tuberculosis,* no. (%), n = 5,849
Age			
0–19	3 (9.7)	20 (9.2)	355 (6.1)
20–39	10 (32.3)	38 (17.4)	1,834 (31.4)
40–59	5 (16.1)	49 (22.5)	1,951 (33.4)
60–79	10 (32.3)	70 (32.1)	1,113 (19.0)
80–99	3 (9.7)	41 (18.8)	593 (10.1)
Unknown	0	0	3 (0.1)
Sex			
M	25 (80.7)	143 (65.6)	3,796 (64.9)
F	6 (19.4)	75 (34.4)	2,053 (35.1)
Country of birth			
Other	6 (19.4)	54 (24.8)	2,133 (36.5)
Spain	25 (80.6)	136 (62.4)	2,426 (41.5)
Unknown	0	28 (12.8)	1,290 (22.1)
Primary location			
Pulmonary	16 (51.6)	115 (52.8)	4,460 (76.3)
Extrapulmonary	15 (48.4)	98 (45.0)	1,346 (23.0)
Unknown	0	5 (2.3)	43 (0.7)
HIV laboratory result			
Positive	3 (9.7)	3 (1.4)	368 (6.3)
Negative	18 (58.1)	146 (67.0)	3,713 (63.5)
Unknown	10 (32.3)	69 (31.7)	1,738 (30.2)
Hospitalization			
No	16 (51.6)	60 (27.5)	2,016 (35.4)
Yes	14 (45.2)	145 (66.5)	3,689 (63.1)
Unknown	1 (3.2)	13 (6.0)	144 (2.5)
Treatment outcome			
Complete/cure	26 (83.9)	147 (67.4)	3,563 (60.9)
Abandon/moved/loss	0	5 (2.3)	268 (4.6)
Death	2 (6.5)	27 (12.5)	500 (8.5)
Failure/prolongation	2 (6.5)	7 (3.2)	161 (2.8)
Unknown	1 (3.2)	32 (14.7)	1,357 (23.2)

*Treatment outcome was defined as follows: complete/cure, complete treatment or cure; abandon/moved/loss, abandonment of treatment, relocation, or loss of contact; death, death during treatment; failure/prolongation, treatment failure or patient still in treatment 12 mo after initiation.

**Table 2:** Determinants of *Mycobacterium bovis* infection versus *M. tuberculosis* infection in study of tuberculosis in Spain, 2018–2022*

Category	Crude odds ratio (95% CI)	p value	Adjusted odds ratio (95% CI)	p value
Age, y				
0–19	Referent		Referent	
20–39	**0.37 (0.21–0.65)**	**<0.001**	0.59 (0.26–1.47)	0.28
40–59	**0.45 (0.27–0.78)**	**0.003**	0.67 (0.31–1.62)	0.34
60–79	1.12 (0.68–1.91)	0.7	1.37 (0.64–3.29)	0.45
80–99	1.23 (0.72–2.17)	0.5	1.29 (0.53–3.38)	0.58
Sex				
M	Referent			
F	0.97 (0.73–1.28)	0.8		
Country of birth				
Other	Referent		Referent	
Spain	**2.21 (1.62–3.07)**	**<0.001**	**2.32 (1.44–3.82)**	**<0.001**
Primary infection location				
Pulmonary	Referent		Referent	
Extrapulmonary	**2.82 (2.14–3.72)**	**<0.001**	**2.20 (1.46–3.28)**	**<0.001**
HIV laboratory result				
Positive	Referent		Referent	
Negative	**4.82 (1.82–19.6)**	**0.007**	**3.39 (1.24–14.0)**	**0.04**
Hospitalization				
No	Referent		Referent	
Yes	1.32 (0.98–1.80)	0.075	1.10 (0.72–1.70)	0.66
Treatment outcome†				
Complete/cure	Referent		Referent	
Abandon/moved/loss	0.45 (0.16–1.00)	0.084	0.74 (0.18–2.05)	0.61
Death	1.31 (0.84–1.96)	0.2	1.22 (0.66–2.18)	0.51
Failure/prolongation	1.05 (0.44–2.13)	0.9	0.97 (0.23–2.73)	0.96

*Bold text indicates statistical significance, defined as p<0.05 and an odds ratio with 95% CI not including 1. †Treatment outcome was defined as follows: complete/cure, complete treatment or cure; abandon/moved/loss, abandonment of treatment, relocation, or loss of contact; death, death during treatment; failure/prolongation, treatment failure or patient still in treatment 12 mo after initiation.

The median age of diagnosis for *M. bovis* TB was 60 years (interquartile range [IQR] 37–77 years), higher than that for *M. caprae* (55 [IQR 28–65] years) or *M. tuberculosis* (46 [IQR 32–63] years) (p<0.001, determined by pairwise comparisons of median age at diagnosis across the 3 *Mycobacterium* species, using the Mann-Whitney U test with Bonferroni correction for multiple comparisons). When stratifying by country of origin, we observed significantly higher median age for case-patients infected with *M. caprae* (58 [IQR 28–65] years), *M. bovis* (68 [IQR 53–79] years), and *M. tuberculosis* (55 [IQR 40–73] years) (p<0.001, where p indicates statistically significant differences in median age at diagnosis between patients born in Spain and those born outside of Spain for each *Mycobacterium* species, based on Mann-Whitney U tests with Bonferroni correction for multiple comparisons). For those born elsewhere, median age for case-patients was 35 (IQR 26–44) years for those with *M. caprae*, 38 (IQR 26–62) years for those with *M. bovis*, and 36 (IQR 27–47) years for those with *M. tuberculosis*.

The frequency of pulmonary and extrapulmonary forms was similar for both *M. bovis* and *M. caprae* TB cases; approximately half of the cases had extrapulmonary forms: 98/218 (45%) of those infected with *M. bovis* and 15/31 (48.4%) of those with *M. caprae*. *M. bovis* case-patients were 2.20 (95% CI 1.46–3.28) times more likely to have extrapulmonary TB develop than were *M. tuberculosis* case-patients (Table 2). Of note, we observed a high proportion of lymphatic forms (46/213 [21.6%]) for *M. bovis* and of genitourinary location (7/31 [22.6%]) for *M. caprae* (Appendix Table 1).

The percentage of HIV co-infection was 9.7% (3/31 [95% CI 3.3–24.9]) in *M. caprae* cases, compared with 1.4% (3/218 [95% CI 0.5–4.0]) in *M. bovis* cases. *M. bovis* case-patients were 3.39 (95% CI 1.24–14.0) times more likely to be HIV negative than were *M. tuberculosis* case-patients.

Two-thirds of *M. bovis* and *M. tuberculosis* case-patients were hospitalized: 145/218 (66.5% [95% CI 60.0%–72.4%]) of case-patients with *M. bovis* infection and 3,689/5,849 (63.1% [95% CI 61.8%–64.3%]) of those with *M. tuberculosis*. Less than half (14/31; 45.2% [95% CI 29.2%–62.2%]) of *M. caprae* TB case-patients were hospitalized. More than three-quarters (26/31; 83.9% [95% CI 67.4%–92.9%]) of *M. caprae* TB case-patients completed treatment, followed by approximately two thirds of *M. bovis* (147/218; 67.4% [95% CI 60.9%–73.3%]) and *M. tuberculosis* (3,563/5,849; 60.9% [95% CI 59.7%–62.1%]) case-patients (Table 1).

### Temporal Trends

**Figure 1:**
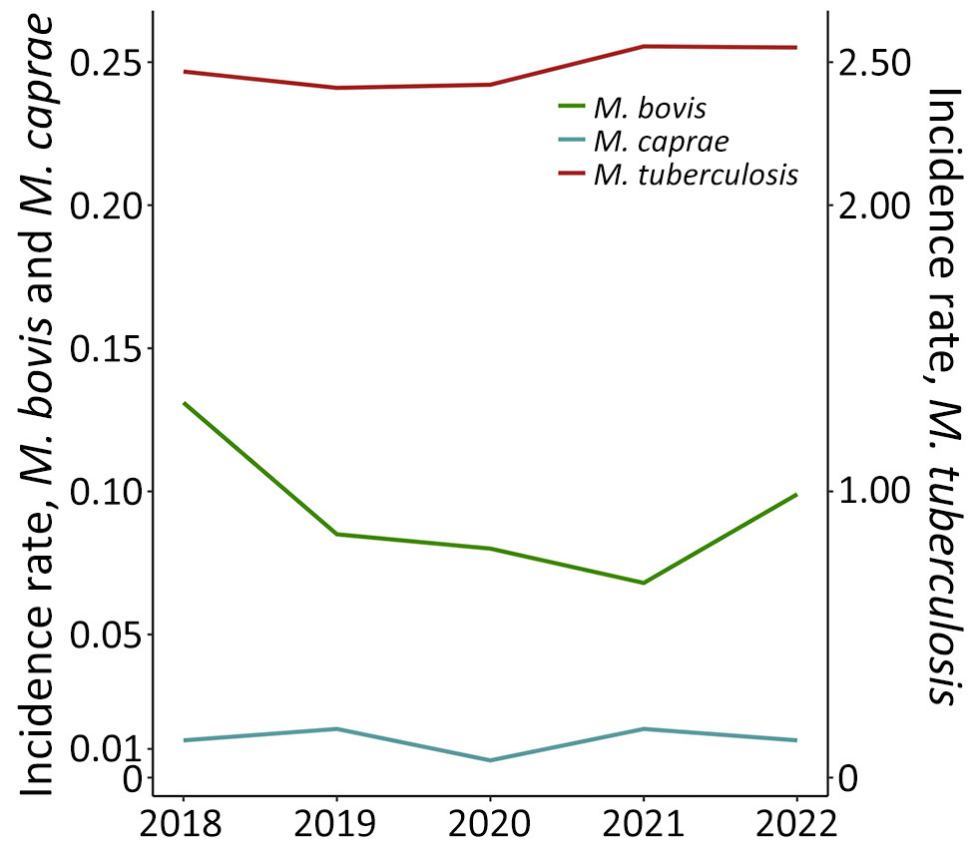
Incidence rates of tuberculosis cases per 100,000 population by causative agent and year of notification, Spain, 2018–2022. Scales for the y-axes differ substantially to underscore patterns but do not permit direct comparison.

The mean incidence rate per 100,000 population in 2018–2022 was 0.092 for *M. bovis*, 0.013 for *M. caprae*, and 2.48 for *M. tuberculosis*. TB cases notified with *M. tuberculosis* as a causative agent increased in 2021 and remained stable in 2022 (Figure 1; Appendix Table 2). The statistical trend analysis by joinpoint regression revealed no significant annual percentage change of *M. bovis* and *M. caprae* incidence.

### Geographic Distribution and Spatial Analysis

**Figure 2:**
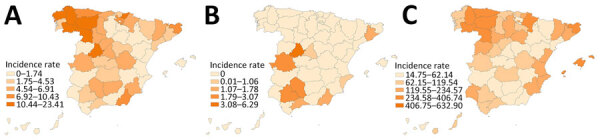
Incidence of tuberculosis reported by province, Spain, 2018–2022. A) *Mycobacterium bovis*; B) *M. caprae*; C) *M. tuberculosis*. Incidence rates for regions are per 1 million population.

We observed higher rates of *M. bovis* in north and northwestern Spain. *M. caprae* cases were reported in the central and southern part of Spain and the province of Barcelona; 9 of 54 provinces reported human cases of *M. caprae* during 2018–2022 (Figure 2).

**Figure 3:**
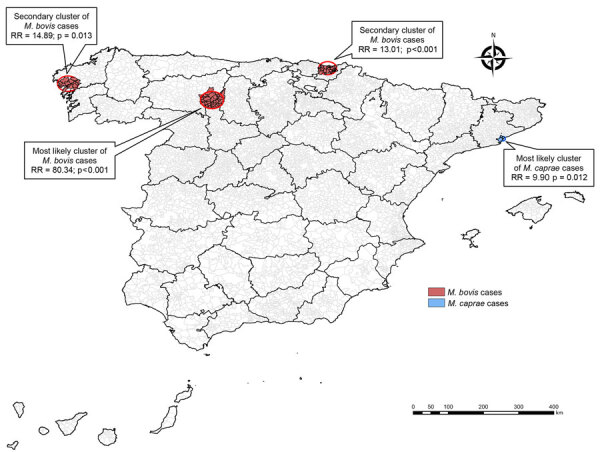
Spatial clusters of tuberculosis infections caused by *Mycobacterium bovis* and *M. caprae*, Spain, 2018–2022. RR, relative risk.

We detected 3 notable spatial clusters of *M. bovis* and 1 of *M. caprae* cases (Table 3; Figure 3). The most likely cluster of *M. bovis* was located in the eastern part of the province of León. Two other clusters with a significantly higher number of cases observed than expected were located in the coastal area of the Basque Country (relative risk [RR] 13.01; p<0.001) and the west part of Galicia (RR 14.89; p = 0.013). The only significant spatial cluster of *M. caprae* TB cases was situated in the Barcelona metropolitan area (RR 9.90; p = 0.012) (Table 3; Figure 3). All case-patients from *M. bovis* clusters were native born older adults (median age 75 [IQR 66–86] years), mostly men (16/24, 63%), and all HIV-negative; 67% (16/24) had pulmonary manifestations. TB case-patients from the *M. caprae* cluster were all native born and mostly older men (median age 72 [IQR 65–74] years); 7/9 had genitourinary manifestations.

**Table 3:** Spatial clusters of *Mycobacterium bovis* and *M. caprae* cases, Spain, 2018–2022

Cluster	No. municipalities	Radius, km	No. observed cases	No. expected cases	Relative risk	p value
*M. bovis*						
1, most likely	38	24.4	6	0.77	80.34	<0.001
2	34	18.5	11	0.89	13.01	<0.001
3	13	21.8	7	0.48	14.89	0.013
*M. caprae*						
1	5	6.4	9	1.26	9.90	0.012

## Discussion

This study describes characteristics and spatiotemporal distribution of human TB cases caused by *M. bovis* and *M. caprae* bacteria at the national level in Spain over a 5-year period. First, we aimed to describe and compare the population and clinical characteristics of the infections by different MTBC species, which may contribute to better understanding of the epidemiology of the disease. We found differences in age, country of birth, primary location of infection, and HIV status in *M. bovis*, *M. caprae*, and *M. tuberculosis* cases. Although no significant sex differences were detected, case-patients were predominantly male for all 3 MTBC species, especially for *M. caprae* infection. Higher male-to-female ratio was previously described in Spain and other countries in Europe in older adults, which has been related to different behavioral and environmental risks, lifestyle, or biological factors (*6*,*16*). Native-born case-patients were significantly older than foreign-born case-patients for all 3 MTBC species; the age difference was more pronounced for *M. bovis* cases, which could suggest reactivations rather than new infections. A similar scenario of possible reactivation of latent infections has been suggested in the United Kingdom and Italy, where *M. bovis* cases occurred predominantly in the elderly and native-born (*17*,*18*). However, recent infections associated with occupational exposure cannot be ruled out; Palacios et al. demonstrated that in a region of low incidence of bovine TB in Spain, half of patients >45 years of age shared the genotype with circulating cattle strains, suggesting a recent transmission (*19*).

*M. bovis* and *M. caprae* case-patients in our study were mainly native-born and had an extrapulmonary infection, in contrast to *M. tuberculosis* case-patients, corroborating previous results in Europe and the United States (*20*,*21*). Those differences could also be attributed to consuming unpasteurized dairy products as the main route of transmission of zTB. Lymph nodes and genitourinary system, followed by bones and joints, were the most common primary locations of extrapulmonary TB in *M. bovis* and *M. caprae* cases, as previously described for zTB cases (*20*,*22*). The high proportion of extrapulmonary forms observed in *M. bovis* and *M. caprae* cases may hinder the detection and diagnosis; therefore, it is essential to improve the knowledge of the clinical manifestations caused by zTB species to contribute to raising awareness and guiding clinicians’ diagnosis and treatment.

HIV co-infection was more common in *M. tuberculosis* than *M. bovis* cases, which could be associated with younger patient age and a more urban profile of *M. tuberculosis* cases (*23*). The low number of HIV infections among *M. caprae* cases made it difficult to draw conclusions. More than two thirds of *M. bovis* and *M. tuberculosis* case-patients were hospitalized; in contrast, less than half of *M. caprae* were hospitalized. Those differences could be related to the higher age observed in *M. bovis* cases or the less severe extrapulmonary manifestations observed for *M. caprae*. Moreover, we did not observe differences in treatment outcome among MTBC species, but the limited completeness of the information collected for this variable limited its interpretation.

We did not observe a notable upward or downward temporal trend in the incidence rate during the study period for *M. bovis* or *M. caprae* cases. Similar figures were reported by EFSA and ECDC at the Europe level; Spain accounted for one third of the cases of *M. bovis and M. caprae* (*3*). We observed no decline in median age of native-born *M. bovis* and *M. caprae* cases over the study period, as previously observed in the United Kingdom during 2014–2022 (*24*). It was suggested that our findings might be related to increased consumption of unpasteurized milk; however, per capita raw milk consumption has been in steady decline since the 1990s in Spain, with the exception of the COVID-19 pandemic in 2021–2022 (*25*).

The geographic distribution indicated a pattern of higher incidence in northwest Spain for *M. bovis* and *M. tuberculosis*, whereas *M. caprae*, cases were reported in the central and southern part of Spain and the province of Barcelona. This pattern contrasted with the reported incidence of TB in bovines (*12*); northwest Spain is where most of the officially TB-free provinces, in which cattle farms are mainly for dairy animals, are concentrated. In contrast, the central-western and southern regions have the highest incidence, where most of the beef cattle farms are located. For *M. caprae* infections, the distribution of human cases and the prevalence and population density in goats shows some overlap, as described in an integrative genomic analysis of human and goat strains in Andalusia (*26*). Three spatial clusters of *M. bovis* cases were detected in bovine TB–free regions of the northern and northwestern part of Spain, affecting mainly older native-born men with pulmonary tuberculosis. However, those clusters were located in historically milk-producing regions of northern Spain, 2 of them in a humid area with an oceanic climate (*27*), whereas higher incidence of infection has been negatively associated with sunshine exposure and vitamin D levels (*28*). The *M. caprae* cluster also affected mainly older native-born persons, but conversely to *M. bovis* clusters, it was located in a large metropolitan area and manifested as genitourinary presentation. The geographic distribution of zTB cases observed together with the characteristics of the patients could support the previously described hypothesis of reactivation of an old infection. Our study of the epidemiology of zTB in Spain is part of a larger project that will include a genomic analysis of human strains and those circulating in cattle and goats to confirm our findings and to establish epidemiologic links and zoonotic transmission.

We note that we did not have information on occupational activities and other individual risk factors or medical history for case-patients. We used place of residence, which does not have to correspond to the place of exposure, for the spatial cluster analysis. In addition, 32.3% of reported TB cases had data on MTBC species, which could have led to an underestimation of *M. bovis* and *M. caprae* cases; the low rate is likely because most TB laboratories identify mycobacteria at the level of the MTBC and do not differentiate between species or do not report the MTBC species. Zoonotic cases account for only a small proportion of TB cases; clinical management is the same, and additional molecular methods not available in all laboratories are required to distinguish species. Efforts to improve completeness of information on MTBC species have included assessment reports sent to the CCAA to retrieve information, as well as investments in the information systems interoperability; we were able to recover unreported zTB cases through collaboration with the CCAA.

The findings from our study of the clinical and demographic characteristics and spatiotemporal distribution of the different zTB cases in Spain can support future genomic epidemiology studies that include data on circulating strains in humans and animals. Understanding the epidemiology of the human cases of *M. bovis* and *M. caprae* and the underlying mechanisms of transmission can contribute to the prevention and control of zoonotic outbreaks. Our study also highlights the need for improved integrated epidemiologic and laboratory information, in particular on circulating MTBC species. We recommend the close and active collaboration of public and animal health institutions to contribute to zTB control and eradication.

Appendix Additional information about spatiotemporal distribution and clinical characteristics of zoonotic tuberculosis, Spain, 2018–2022.
