# Correction: Vol. 31, No. 7

**DOI:** 10.3201/eid3108.C13108

**Published:** 2025-08

**Keywords:** errata, erratum, correction

The name of author Evangelia Ouranou was incorrect in Spatiotemporal Distribution and Clinical Characteristics of Zoonotic Tuberculosis, Spain, 2018–2022 (Á. Roy et al.). The article has been corrected online (https://wwwnc.cdc.gov/eid/article/31/7/25-0031_article).

