# Supplementary material for: Spatiotemporal Distribution and Clinical Characteristics of Zoonotic Tuberculosis, Spain, 2018–2022
Source: Emerg Infect Dis. 2025 Jul;31(7):1344–52. doi: 10.3201/eid3107.250031 (PMC12205449; doi:10.3201/eid3107.250031)
Supplement: Appendix — Additional information about spatiotemporal distribution and clinical characteristics of zoonotic tuberculosis, Spain, 2018–2022. [file 25-0031-Techapp-s1.pdf]

Article DOI: https://doi.org/10.3201/eid3107.250031

EID cannot ensure accessibility for supplementary materials supplied by authors. Readers who have difficulty accessing supplementary content should contact the authors for assistance.

Spatiotemporal Distribution and Clinical Characteristics of Zoonotic Tuberculosis, Spain, 2018–2022

Appendix

Appendix Table 1. Primary location of confirmed TB patients with *M. caprae*, *M. bovis,* and *M. tuberculosis* between 2018–2022*

Primary location	*M. bovis,* no. (%)	*M. caprae*, no. (%)	*M. tuberculosis*, no. (%)
Pulmonary	115 (54.0)	16 (51.6)	4,460 (76.8)
Digestive	6 (2.8)	1 (3.2)	57 (1.0)
Disseminated	2 (0.9)	1 (3.2)	69 (1.2)
Genitourinary	13 (6.1)	7 (22.6)	92 (1.6)
Lymphatic	46 (21.6)	3 (9.7)	544 (9.4)
Meningeal	2 (0.9)	0 (0.0)	30 (0.5)
Osteoarthritic	13 (6.1)	1 (3.2)	132 (2.3)
Other organs	11 (5.2)	1 (3.2)	83 (1.4)
Pleural	5 (2.3)	1 (3.2)	339 (5.8)

*Missing values: *M. bovis* (n = 5), *M. tuberculosis* (n = 43).

Appendix Table 2. TB incidence rates due to *M. bovis, M. caprae, and M. tuberculosis* between 2018–2022 per 100,000 inhabitants

Year	*M. caprae* cases	Incidence rate of *M. caprae* per 100,000 inhabitants	*M. bovis* cases	Incidence rate of *M. bovis* per 100,000 inhabitants	*M. tuberculosis* cases	Incidence rate of *M. tuberculosis* per 100,000 inhabitants
2018	6	0.013	61	0.131	1151	2.467
2019	8	0.017	40	0.085	1131	2.410
2020	3	0.006	38	0.080	1146	2.421
2021	8	0.017	32	0.068	1211	2.555
2022	6	0.013	47	0.099	1210	2.551
2018–2022	31	0.013	218	0.092	5849	2.481
